# Factors associated with eye disorders and diseases: A retrospective study

**DOI:** 10.12669/pjms.41.1.9728

**Published:** 2025-01

**Authors:** Olgun Goktas

**Affiliations:** 1 Olgun Goktas, Associate Professor, Uludag University Family Health Center, Nilufer, Bursa, Turkey

**Keywords:** Associated factors, Disorders, Diseases, Eye, Family medicine practice

## Abstract

**Objective:**

To retrospectively identify the factors associated with eye disorders and diseases.

**Methods:**

The retrospective study was carried out in Bursa Uludag University Family Health Center in Turkey between 1-30 September 2023. The data of individuals who were registered with the Family Health Center and whose eye disorders and diseases were known were evaluated retrospectively. Eye disorders and diseases, sociodemographic findings, existing diseases, allergies, cancer, genetic disease conditions, and steroid use were obtained.

**Results:**

Around 3658 people; 1849 males (50.5%) and 1809 females (49.5%) participated in the study. The mean age of the individuals was 26.98±10.22 years. It was determined that 1594 (43.6%) of the participants had at least one eye disorder and/or disease and 1511 (41.3%) wore glasses and/or contact lenses. In 1594 (43.6%) patients with eye disorders or diseases, 41% had refractive disorders. It was determined that the rate of eye diseases is higher in women, smokers, and obese people, those with a family history of eye diseases, those with allergic and chronic diseases, those with genetic diseases, and those who use steroids (p=0.04).

**Conclusion:**

It is noteworthy in terms of eye health that nearly half of the participants have at least one eye disorder and/or disease, and nearly half of them use glasses and/or contact lenses. It is also important that eye disorders and diseases are seen more together with smoking, obesity, allergies, genetic and chronic diseases, and steroid use. We emphasize the holistic approach of family medicine and the importance of cooperation with other clinics in terms of eye health.

## INTRODUCTION

Most eye diseases and vision disorders can be prevented with early diagnosis and treatment. Today, the increase in eye disorders and diseases in parallel with the increase in the world population and other diseases increases the need for health care for eye health. With good care and preventive medicine, eye diseases and disorders can be prevented and treated before they become chronic and complications develop. In a meta-analysis study conducted across many clinical branches, it was shown that blindness due to eye diseases such as refractive errors, glaucoma, and cataracts can be prevented before they cause blindness with early diagnosis and treatment.^1^

Although diagnosis and treatment opportunities are improving with technology, complications related to eye diseases and disorders lead to permanent diseases, disabilities, and decreased quality of life in individuals. In the studies conducted in parallel with the Global Disease, Injury and Risk Factors (GBD) 2019 Study, it was determined that eye disorders and diseases are increasing. Although an effective policy is followed in the fight against eye diseases and blindness, it is recommended to raise public awareness to reduce especially severe visual impairments.^2^,^3^

The eye is a multi-component organ and has a complex structure. Due to the eye’s relationship with other organs and body structure, other diseases may cause symptoms in the eye. Due to this feature, symptoms related to diseases and disorders in the eye organ may occur not only from the eyeball but also from other diseases. In a study, it was noted that people with ocular diseases, especially those using psychotropic drugs, had at least one psychiatric symptom.^4^ Similar studies emphasize that the prevalence of visual impairment and eye diseases is increasing, but most of these diseases can be prevented earlier with preventive medicine.^5^-^7^

Large-scale eye care services are needed to cope with the increase in preventable blindness and vision loss, where surgically correctable cataracts and glasses-corrected refractive errors continue to increase in those aged 50 and over.^8^ A meta-analysis study found that global eye care services are due to the alarming increase in age-related macular degeneration. It is recommended that strategies and healthcare models be created urgently.^9^ In a systematic analysis conducted by age, geographical region, and year, it is emphasized that eye disorders and diseases vary greatly and that this will be effective in eye care planning and allocation.^10^ In another study, blindness and low vision are stated that it is generally caused by chronic eye disorders and diseases over the age of 50.^11^ The risk of death from all causes was higher in those with visual impairment than in those with normal vision, depending on the severity of the visual impairment.^12^

In family medicine, holistic and continuous health care that evaluates the individual and family as a whole is important in preventing eye diseases and disorders. Evaluation of eye diseases requires not only the ophthalmology clinic but also all other clinical branches and healthcare professionals. Especially in terms of preventive medicine and follow-up, it is appropriate to coordinate individuals with eye symptoms in family medicine first. Evaluation of registered individual data in family medicine is important in terms of improving the missing information in the literature on eye diseases. In this study, we aimed to identify the factors, which may be associated with eye diseases and disorders, detected within the specified period in individuals registered at the family health center.

## METHODS

This retrospective study was carried out at Bursa Uludağ University Family Health Center between 1-30 September 2023. The data of individuals registered in the Family Health Center and whose eye disorders and diseases (recorded in the Family Medicine Information Registration System) were known were evaluated retrospectively. Patient records, which are secondary data sources, were used as a data collection method. Individual data such as age, gender, educational status, smoking, alcohol use, cancer, allergy, genetics, chronic illnesses, steroid use, use of glasses and/or contact lenses, and any eye disorder and disease during the research period were obtained from the family medicine information registration system.

The categories of eye diseases of the patients included in the study were considered as visual disorders, diseases caused by infection, and diseases caused by trauma-acute situations. In addition, diseases other than these classifications were examined under the other group.

There are some limitations and assumptions in the study. Firstly, the study was conducted in a university family health center serving a specific region. The study was conducted on N = 3658 patients who were admitted to the family health center in September 2023 and whose data were sufficient. The demographic and clinical characteristics of this group are thought to be very important in the emergence of the current results. It is thought that changing patient profiles may change the results, and it is important to evaluate the results obtained depending on this limitation. It is also quite possible that the frequency of certain diseases will increase or decrease seasonally during the relevant period. Finally, it is thought that the disease groups determined at the basic level, such as visual disorders, infection-related, and trauma-acute disease groups, have homogeneous characteristics within themselves. In addition, the assumptions of the study include that the data obtained from the family health center database is accurate and that doctors assign correct diagnoses to patients.

While determining the study sample, the sample calculation was made based on the annual number of patients in the family health center. It has been determined from the records that the number of patients in the family health center in 2023 is approximately 36,000. With the simple random sampling method, it was determined that at least n=1648 patients should be included in the study at a 1% error and 95% confidence level. 3658 Patients whose data were recorded in the database and whose records were compatible with the actual information in the patient file were included in the study. It can be seen that the sample’s representation of the universe is high.

### Ethical approval:

The study was carried out after approval from the Clinical Research Ethics Committee of Bursa Uludağ University, Faculty of Medicine (Ref. dated 2023-08-01/ decision no:2023-16/12) following the Declaration of Helsinki.

### Statistical analysis:

In the study, descriptive statistics of individuals are given with mean, standard deviation, frequency, and percentage values. Chi-square analysis was applied to examine the demographic and clinical characteristics of the patients according to the presence of complementary eye disease. Independent sample t-test analysis was performed to examine age measurements according to the presence of complementary eye disease. The critical decision-making level for p-Value was < 0.05. SPSS (Statistical Package for Social Science, Chicago, II, USA) 25.0 Windows package program was used for statistical evaluation.

## RESULTS

A total of 3658 people, 1849 men (50.5%) and 1809 (49.5%) women, participated in the study. It was determined that the average age of the individuals was 26.98±10.22 (mean±SD). It was also observed that the rate of smoking among individuals was 2.9% and the rate of alcohol use was 0.5% and 8.5% of individuals were obese.

It also revealed that the rate of having a family history of eye disease was 6.8%. It was determined that 0.3% of the individuals had a history of eye trauma and 1.3% of the individuals had a history of eye surgery. It was found that 3.7% of the individuals had genetic diseases, 1.5% had cancer, 9.6% had allergic diseases and 22.6% had chronic diseases. It was also noted that 1.9% of the participants used steroids. 41.3% of individuals use glasses or contact lenses while 1594 (43.6%) of the 3658 registered individuals had at least one eye disorder and/or disease, (Table-I).

**Table-I:** General characteristics of the participants.

Parameters		n	%	
Sex	Male	1849	50.5%	
	Female	1809	49.5%	
Age	*χ̅*±*S* (mean±SD)	26.98±10.22		
Education	High school and before	331		9.0%
	University student	2319		63.4%
	Graduated from a university	1008		27.6%
Smoking	No	3553	97.1%	
	Yes	105	2.9%	
Alcohol	No	3641	99.5%	
	Yes	17	0.5%	
Obesity	No	3348	91.5%	
	Yes	310	8.5%	

Of the 1594 patients with eye disorders or diseases, 41% had refractive disorders, 5.9% had corneal diseases (Dry eye, etc.), 4.3% had eye infections, 0.4% had strabismus (Amblyopia, etc.), 0.3% had glaucoma, 0.2% had retinal diseases (Age-related macular degeneration, etc.), and 0.2%, and other eye diseases (optic neuritis, eye trauma, tumor, blindness, etc.) were found. There was only one patient (0.0%) with cataract who had not yet been treated, (Table-II).

**Table-II:** Clinical characteristics of the participants.

Etiologies		n	%
Eye disease in the family	No	3409	93.2%
	Yes	249	6.8%
Eye Trauma	No	3646	99.7%
	Yes	12	0.3%
Eye Surgery	No	3611	98.7%
	Yes	47	1.3%
Genetic disease	No	3522	96.3%
	Yes	136	3.7%
Cancer	No	3602	98.5%
	Yes	56	1.5%
Allergy	No	3306	90.4%
	Yes	352	9.6%
Chronic Disease	No	2830	77.4%
	Yes	828	22.6%
Steroid Use	No	3589	98.1%
	Yes	69	1.9%
Use of glasses and/or contact lenses	No	2147	58.7%
	Yes	1511	41.3%
Presence of Eye Disease	No	2064	56.4%
	Yes	1594	43.6%

We also noted that the incidence of eye diseases varies depending on the gender of individuals. It was observed that the incidence of eye diseases was higher in women than in male patients (p = 0.01). The incidence of eye diseases varies depending on individuals’ smoking status. It was observed that the incidence of eye diseases was higher in smokers (p = 0.04). It has been determined that the incidence of eye diseases varies depending on the obesity status of individuals. It was noted higher in those who were obese (p = 0.04). It has been determined that the incidence of eye diseases varies depending on the age of individuals. The patients’ eye disease presence was significantly different according to their etiology groups. We also noted that eye disease in refractive disorders group patients was significantly higher than in other groups (p= 0.01). We also noted that the ages of individuals with the disease were higher than those without the disease (p = 0.01), (Table-III).

**Table-III:** Type of Eye Disease.

Etiologies	n	%
Refractive disorders	1500	41.0%
Corneal diseases	216	5.9%
Eye infections	157	4.3%
Other eye diseases (Optic neuritis, trauma, tumor, blindness, etc.)	21	0.6%
Strabismus	16	0.4%
Glaucoma	11	0.3%
Retinal diseases	8	0.2%
Cataract	1	0.0%

It has been determined that the incidence of eye disease varies depending on whether individuals have a family history of eye disease. The incidence of eye disease was higher in those with a family history of eye disease (p = 0.01). It has been determined that the incidence of eye diseases varies depending on whether individuals have a history of genetic disease. It also depends that the incidence of eye diseases was higher in those with genetic diseases (p = 0.03). We also found that the incidence of eye disease did not differ depending on whether individuals had a history of cancer (p=0.08). The incidence of eye disease varies depending on whether individuals have a history of allergic disease.

We observed that the incidence of eye disease was higher in people with allergic diseases (p = 0.01). It has been determined that the incidence of eye diseases varies depending on whether individuals have a history of chronic disease. It was observed that the incidence of eye diseases was higher in those with chronic diseases (p=0.01). It has been determined that the incidence of eye disease varies depending on individuals’ steroid use. The incidence of eye diseases was higher in patients using steroids (p=0.04). It has been determined that the incidence of eye diseases varies depending on whether individuals use glasses or contact lenses. In the study, it was observed that the incidence of eye diseases was higher in individuals using glasses or contact lenses (p=0.01), (Table-IV).

**Table-IV:** Examination of the Presence of Eye Disease According to Patient Characteristics.

		Presence of eye disease				p

		No		Yes		

		n	%	n	%	
Sex***	Male	1115	54.0%	734	46.0%	0.01*
	Female	949	46.0%	860	54.0%	
Education***	High school and before	241	11.7%	90	5.6%	0.09
	University student	1313	63.6%	1006	63.1%	
	Graduated from a university	510	24.7%	498	31.2%	
Smoking***	No	2063	100.0%	1490	93.5%	0.04*
	Yes	1	0.0%	104	6.5%	
Alcohol***	No	2064	100.0%	1577	98.9%	-
	Yes	0	0.0%	17	1.1%	
Obesity***	No	2020	97.9%	1328	83.3%	0.01*
	Yes	44	2.1%	266	16.7%	
Etiologies***	Refractive disorders	101	96.2%	1493	79.3%	0.01*
	Corneal diseases	1	1.0%	215	11.4%	
	Eye infections	2	1.9%	155	8.2%	
	Others	1	1.0%	20	1.1%
Age**	X±SD (mean±SD)	24.81±6.62		29.78±13.01		0.01*

*** Chi-square analysis,

** t test analysis

* Significant difference at 0.05 level.

## DISCUSSION

Nearly half of the individuals in our region’s population (43.6%) had at least one eye disorder and/or disease, and nearly half of them (41.3%) wore glasses and/or lenses. Among those with eye disorders or diseases, refractive disorders were most prevalent in 41% of cases. These results are important for all societies as well as the registered population within family medicine practice. The eye is an important human organ. The results of this study are valuable in terms of showing the rate of eye health and diseases in society.

The first place where eye disorders and diseases are usually diagnosed is primary health care institutions. It has been reported that 77% of eye disorders detected incidentally during pre-work eye screening in a tertiary eye hospital are asymptomatic.^13^ In studies including hospital results where color vision deficiency screening and pediatric eye checks are performed, it is reported that vision screenings and initial eye examinations should be performed in primary healthcare institutions.^14^,^15^ In our study, 63.4% of the participants were university students and 63.1% of these students had at least one eye disease, mostly refractive disorders.

A study conducted on adults in China reported that failure to provide the necessary corrective treatment in those with visual impairment resulted in a worse lifestyle and early death. In this study, detection and treatment of visual disorders and eye diseases at an early age is recommended.^16^ In our study, the average age of 1594 patients with eye diseases was 24.81±6.62 years, and these patients constituted 43.6% of all participants. This rate is high, and we would like to emphasize the importance of detecting visual impairments and eye diseases at an early age and following up individuals with a family physician with continuous, comprehensive care.

In our study, refractive disorders were detected in 41% of 1594 patients with eye disorders or diseases. A study similar to the results of our study mentions uncertainty regarding the increase in the prevalence of myopia and high myopia in the near future until 2050. It is stated that myopia is an important cause of vision loss and that appropriate services should be planned due to its prevalence and increase worldwide.^17^ Similarly, it is stated that the prevalence of refractive errors depends on eye care services as well as age, gender, ethnicity, geographical region, and awareness programs are recommended, especially among young adults.^18^ According to our study, it is important to design and implement such programs in family medicine.

We determined that 22.6% of the patients with eye disease had at least one chronic disease. In one study, it is recommended that early brain imaging be performed without delay in cases where hearing loss and tinnitus occur behind visual impairment.^19^ About 60% of visually impaired school children have psychiatric diseases at some point in their lives^20^, and another study states that pseudomyopia should be distinguished from psychiatric symptoms.^21^ We emphasize the importance of preventive medicine in family medicine with a holistic approach to distinguishing eye disorders and psychiatric diseases from eye diseases.

In one study, it was stated that non-genetic factors may play a role in the etiology of eye and vision disorders in congenital and hereditary anomalies.^22^ In our study, the fact that 3.7% of the patients with eye disorders and diseases had a The genetic disease was significant. In our study, it was found that the incidence of eye disease was higher in patients with genetic diseases. Some medications used in dermatology can cause ocular side effects. In a study reporting that steroid treatment causes steroid glaucoma, cataract and permanent optic nerve damage, cooperation between physicians is recommended.^23^ We found that the incidence of eye diseases was higher in patients using steroids.

In a review, it is recommended that due to the relationship of keratoconus with allergy and frequent eye rubbing, allergy should be controlled by measuring IgE if possible.^24^ In our study, the incidence of eye disease was significantly higher in those with allergic diseases. A study emphasizes that there is insufficient awareness of smoking, which is a risk factor for various eye disorders such as cataracts and age-related macular degeneration.^25^ We also noted that the incidence of eye diseases was higher in patients who smoked.

Obesity is known as an important risk factor for many eye diseases. In a review, more strategic studies are needed for the mechanisms that cause eye disease and it is recommended to encourage weight loss in obese people to protect from eye complications.^26^ The incidence of eye disease was found to be higher in obese people.

The study showed that women had higher levels of eye diseases than men. Similarly, comparisons have shown that smoking, alcohol, obesity and advanced age are factors that increase the risk of eye diseases. Previous studies in many different fields have shown that different diseases have different prevalence levels according to patient characteristics in the studies examined.^27^-^32^

Previously reviewed studies have shown that women have higher levels of anxiety than men^27^, and men are more prone to cardiovascular diseases, especially after the age of 40.^28^ It has been stated that smoking and drinking alcohol may similarly cause cardiological diseases.^29^,^30^ Similarly, male patients have been found to have a higher risk of obesity and diabetes than females.^31^,^32^

We were able to obtain results regarding how frequently eye disorders and diseases are seen in our population and to what extent they occur together with the disease and sociodemographic characteristics. It also provided new information regarding risk factors for eye diseases. New studies are needed regarding the relationship between eye diseases and other diseases and conditions.

### Limitations:

Since our study is retrospective, the possibility that some of the results of consultations at other clinics were not included in our records may have created a limitation. Additionally, the frequency of eye diseases and conditions in general was investigated rather than all diseases that may be related to eye health. Systemic relationships with diseases affecting the eye could not be evaluated.

## CONCLUSION

It is noteworthy in terms of eye health that nearly half of the participants have at least one eye disorder and/or disease, and nearly half of them use glasses and/or contact lenses. It is also important that eye disorders and diseases are seen increasingly with smoking, obesity, allergies, genetic and chronic diseases, and steroid use. We emphasize the holistic approach of family medicine and the importance of cooperation with other clinics in terms of eye health.
